# Genomic Landscape of Poorly Differentiated Gastric Carcinoma: An AACR GENIE^®^ Project

**DOI:** 10.3390/life16020209

**Published:** 2026-01-27

**Authors:** Joshua Lodenquai, Tyson J. Morris, Ava Garcia, Emely Sokolovski, Grace S. Saglimbeni, Beau Hsia, Abubakar Tauseef

**Affiliations:** 1 Department of Chemistry, Biola University, 13800 Biola Ave, La Mirada, CA 90639, USA; joshua.lodenquai@biola.edu; 2 Department of Hematology and Oncology, Creighton University School of Medicine, Phoenix, AZ 85012, USA; tysonmorris@creighton.edu (T.J.M.); gracesaglimbeni@creighton.edu (G.S.S.); beauhsia@creighton.edu (B.H.); 3 Department of Biology, Santa Clara University, 500 El Camino Real, Santa Clara, CA 95053, USA; ahgarcia@scu.edu; 4 College of Biological Sciences, University of California, 605 Hutchison Drive, Davis, CA 95616, USA; 5 Division of Hematology and Oncology, Department of Internal Medicine, Creighton University Medical Center, Omaha, NE 68124, USA

**Keywords:** poorly differentiated gastric carcinoma, somatic mutation profiling, genomic co-occurrence, metastatic gastric cancer, mutual exclusivity

## Abstract

Poorly differentiated gastric carcinoma (PGC) is aggressive, yet subtype-specific genomics are under-characterized. We queried AACR Project GENIE^®^ (cBioPortal v18.0-public; 12 August 2025) for PGC and analyzed somatic alterations from targeted panels (depth ≥ 100×; variant allele frequency ≥ 5%). Mutation and copy number frequencies were summarized, co-occurrence and exclusivity were tested, and primary versus metastatic tumors were compared using chi-square with Benjamini–Hochberg correction. The cohort included 189 tumors from 188 patients (71% primary; 25% metastatic), with primary and metastatic tumor samples being collected from different patients. Recurrently mutated genes were *TP53* (48.7%), *CDH1* (31.2%), *ARID1A* (21.2%), *KMT2C* (8.5%), and *POLD1* (7.4%); additional alterations involved *ERBB3*, *KMT2D*, *KEL*, *CDKN2A*, and *FAT1* (≈1–7%). Amplifications in *CCNE1* (8.2%) and *FGFR2* (7.6%) were common, alongside gains in *MET*, *MYC*, *KRAS*, and *ERBB2* and losses in *CDKN2A/CDKN2B*, *CDH1*, and *PTEN*. Significant co-occurrence was observed for *POLD1–KMT2D* (*p* < 0.001), *POLD1–ARID1A* (*p* < 0.001), and *ARID1A–KMT2D* (*p* < 0.001), while *TP53* was mutually exclusive with *ARID1A* (*p* = 0.029) and *CDH1* (*p* = 0.041). *CDH1* (48.9% vs. 29.6%; *p* = 0.021) and *MLH1* (8.5% vs. 1.5%; *p* = 0.040) were enriched in metastases, and *CCNE1* alterations showed female predominance (*p* = 2.83 × 10^−4^). Several “primary-only” findings likely reflect small denominators and require replication. PGC demonstrates a mutational framework dominated by *TP53*, *CDH1*, *ARID1A*, and recurrent *CCNE1*/*FGFR2* amplifications, underscoring dysregulation of cell cycle and chromatin-remodeling pathways as key drivers. Co-occurrence of *POLD1*, *ARID1A*, and *KMT2D* suggests coordinated disruption of DNA repair and epigenetic regulation, whereas mutual exclusivity of *TP53*, *ARID1A*, and *CDH1* indicates distinct tumorigenic routes. Metastatic enrichment of *CDH1* and *MLH1* supports their roles in invasion and therapeutic resistance. Together, these findings highlight candidate biomarkers and actionable pathways warranting validation in larger, multi-omic cohorts to refine precision treatment strategies for this aggressive gastric cancer subtype.

## 1. Introduction

Poorly differentiated carcinoma of the stomach (PGC)—also known as poorly differentiated or undifferentiated gastric adenocarcinoma—is an aggressive gastric cancer subtype marked by rapid proliferation and loss of normal gastric architecture [1,2,3]. It typically arises in the gastric mucosa with minimal or no glandular differentiation, often following long-standing atrophic gastritis [1,3]. Histologically, PGC may appear as tubular, signet-ring cell, or mucinous adenocarcinomas, though grading primarily applies to tubular types [1,3]. According to the Laurén classification, PGCs are categorized as diffuse or intestinal, with poorly differentiated components often found at the invasive margins of intestinal-type tumors [1,3]. Clinically, PGCs present at late stages with deep invasion and frequent lymph node involvement [4]. Early-stage gastric cancer is often asymptomatic, though ~50% experience dyspepsia [1,3]. Advanced disease may manifest with abdominal pain, hematemesis, vomiting, anorexia, and weight loss. Survival correlates with tumor stage and size: 5-year survival is ~67% overall but falls to 14% for tumors ≥10 cm [4,5]. Increased nodal involvement also worsens prognosis, dropping 5-year survival from 44% (1–6 nodes) to 11% (15+ nodes) [1,3].

Gastric cancer is globally prevalent, although data specific to poorly differentiated types remain limited. In 1990, gastric cancer was the second most common cancer worldwide, with approximately 800,000 new cases and 650,000 deaths annually–60% of which were in developing countries. Incidence is highest in Eastern Asia, the Andean regions of South America, and Eastern Europe (>40 cases per 100,000 males), and lowest in North America, much of Africa, Southeastern Asia, and Northern Europe. [1] A 2016 study reported gastric cancer as the fifth most common malignancy and the third leading cause of cancer-related death globally [6].

A study reported that 61% of signet-ring cell and mucinous gastric carcinomas were poorly differentiated, with a higher prevalence in women under 60 [4]. Tumorigenesis is driven by epigenetic abnormalities, such as histone modification, microRNA dysregulation, and abnormal DNA methylation [7]. Approximately 8–10% of cases have a familial link, often involving germline mutations in *ATM*, *TP53*, and *BRCA2* [1,3,8]. Risk factors include a high intake of starch, salt, red meat, fat, and N-nitroso compounds, along with smoking, alcohol use, and H. pylori infection (where <0.5% of cases progress to cancer) [1,3,7,9].

Diagnosis of gastric carcinoma is often delayed due to the lack of early symptoms. In Western countries, 80–90% of patients present with advanced disease and poor curability [1,3]. Endoscopy remains the most effective diagnostic tool because of its sensitivity to subtle changes in mucosal color, texture, and architecture—hallmarks of early disease [1,3]. However, early lesions can still go undetected, and multiple jumbo biopsies are often necessary for confirmation. Endoscopically, gastric cancers are classified into growth patterns I, II, and III, which correspond to gross morphological features [1,3]. For advanced disease, the tumor, node, and metastasis (TNM) staging system is used [1,3,10], where tumor describes size, node—spread to lymph nodes, and metastasis—spread to other parts of the body.

Prevention focuses on reducing dietary and environmental risks—such as excess starch, salt, red meat, fat, and N-nitroso compounds—and avoiding smoking and alcohol. H. pylori eradication therapy also lowers the risk of precancerous lesions and gastric cancer [11].

Surgical resection with lymphadenectomy remains the primary treatment [12]. Adjuvant chemo- or radiotherapy is often used in high-risk cases (T3–4, N+), and neoadjuvant therapy may improve surgical outcomes [12]. Diffuse PGCs require wider excision to ensure negative margins [12]. Notably, research has long indicated that poorly differentiated gastric carcinomas may exhibit greater sensitivity to antitumor agents compared to well-differentiated tumors, reinforcing the rationale for optimizing chemotherapeutic strategies [13]. Targeted inhibitors of cytoskeletal components show promise in inducing apoptosis in diffuse PGCs, potentially reducing systemic toxicity by selectively attacking cancer cells, unlike standard chemotherapy, which often damages both malignant and healthy tissues [14,15].

PGCs often involve mutations or loss of heterozygosity (LOH) in tumor suppressors like *TP53* and *E-cadherin* [7,16]. Allelic loss at chromosome 17p, where *TP53* is located, occurs in over 60% of gastric carcinomas and is frequently linked to CpG base transitions [1,7,16]. Specifically, in diffuse-type GCs, reduced or abnormal *E-cadherin* expression correlates with decreased survival [1,3]. Multiple chromosomal loci have been implicated in allelic loss or gain, including 3p, 4, 5q (30–40% near the *APC* locus), 6q, 9p, 17p (>60% near *TP53*), 18q (>60% near *DCC*), and 20q [1,3].

Despite these findings, most molecular studies focus on gastric carcinomas as a broad category, though well-differentiated and poorly differentiated types follow distinct carcinogenic pathways [7]. Gaps in the molecular characterization of specific subtypes are due to technical limitations, tumor heterogeneity, and population variability [17]. Emerging tools such as single-cell RNA sequencing and circulating tumor DNA analysis are enabling more refined classification of gastric cancer subtypes and may facilitate the development of precision-targeted therapies aimed at improving survival outcomes [17].

Despite recent advances, a comprehensive genomic map of poorly differentiated gastric carcinomas (PGCs) is still needed to fully understand the biology of this aggressive cancer. Defining the specific carcinogenic pathways involved may lead to significant breakthroughs in prevention, diagnosis, and treatment. This study aims to characterize the molecular profile of PGCs and uncover their underlying tumorigenic mechanisms through analysis of data from the AACR GENIE^®^ consortium, with the goal of advancing knowledge, informing the development of targeted therapies, and improving diagnostic strategies.

## 2. Materials and Methods

This study received exemption from institutional review board (IRB) oversight by Creighton University (Phoenix, AZ, USA), as it used only de-identified and publicly available data. Clinical and genomic data were obtained from the AACR Project GENIE^®^ database via the cBioPortal platform (v18.0-public) on 12 August 2025. The dataset includes samples archived from 2017 onward.

The AACR Project GENIE database aggregates sequencing data from 19 international cancer centers. All samples were analyzed using targeted gene panels, typically with coverage exceeding 500×.

Each participating institution within the GENIE consortium uses its own bioinformatics pipeline for variant calling and annotation. However, data are standardized according to Genome Nexus protocols. Tools such as GATK help detect the variants, and ANNOVAR (version 2025Mar02) is used to understand their potential impact—though the exact versions of these tools can differ between centers. Even with this effort to standardize things, there can still be differences in how the data are processed—both between and within institutions. While GENIE includes treatment and outcome data for some cancer types, there is no reported treatment information for poorly differentiated stomach cancer.

Patients were included based on a confirmed diagnosis of poorly differentiated gastric carcinoma, identified within a broader cohort of gastrointestinal tumor cases. Tumor samples were categorized as either primary, referring to those obtained from the original gastric tumor site, or metastatic, referring to specimens collected from secondary sites where the cancer had spread. Primary and metastatic samples did not represent matched pairs. To compare gene-specific mutation frequencies between primary and metastatic tumors, chi-squared tests were conducted using the proportion of cases harboring mutations in each group.

The dataset included somatic mutation data, tumor histology, and demographic variables such as age, sex, and race. The sequencing panels weren’t exactly the same at each place, but most of them included important genes tied to gastric cancer, such as *TP53*, *PIK3CA*, and *KMT2D*. Genes not strongly associated with clinical actionability were generally excluded. Additionally, structural variants were not assessed in this study, as they were not consistently captured or reported within the available dataset.

Copy number alterations (CNAs), including homozygous deletions and amplifications, were identified, and their frequencies were calculated to determine common recurrent events. Tumor mutational burden (TMB) was calculated as the number of somatic mutations per megabase of sequenced DNA, including both synonymous and nonsynonymous variants. Raw TMB scores were normalized based on panel size (e.g., 15 mutations in a 1.5 Mb panel = 10 mutations/Mb). These values were further adjusted using regression models from the AACR GENIE consortium to estimate whole-exome sequencing–equivalent TMB, thereby improving comparability across variable panel designs.

Samples with missing or incomplete data were excluded. All statistical analyses were performed in R using RStudio (version 4.5.1, R Foundation for Statistical Computing, Boston, MA, USA). A *p*-value < 0.05 was considered statistically significant. Continuous variables are reported as means ± standard deviations, while categorical variables are presented as frequencies and percentages. Associations between categorical variables were evaluated using the chi-squared test. For continuous variables, data distribution was first assessed for normality; comparisons between two groups were conducted using either a two-sided Student’s *t*-test (for normal distributions) or a Mann–Whitney U test (for non-normal distributions). The Benjamini–Hochberg false discovery rate (FDR) correction was applied to account for multiple comparisons.

Only nonsynonymous somatic mutations—such as missense, nonsense, frameshift, and splice-site variants—were included in the analysis. Variants were required to meet a minimum variant allele frequency (VAF) of 5% and a sequencing depth of at least 100×. Synonymous mutations and variants of uncertain significance (VUS) were excluded. All mutation data were extracted from the harmonized mutation annotation format (MAF) files provided by GENIE, which standardize variant information across contributing centers using concise gene and protein alteration annotations.

## 3. Results

### 3.1. Patient Demographic of PGC

Our analysis included both primary and metastatic samples to provide a comprehensive view of the cohort (Table 1). The final dataset consisted of 189 tumor samples from 188 patients. The majority were female (*n* = 102, 54.3%), while males accounted for 82 cases (43.6%), and sex was unknown for four cases (2.1%). Nearly all participants were adults (*n* = 187, 99.5%), with only one pediatric case (0.5%). By ethnicity, most patients were non-Hispanic (*n* = 136, 72.3%), followed by Hispanic (*n* = 34, 18.1%); ethnicity data were unavailable for 18 patients (9.6%). By race, the majority were White (*n* = 115, 61.2%), followed by Black (*n* = 21, 11.2%) and Asian (*n* = 19, 10.1%). An additional 18 patients (9.6%) were listed as “Other,” and race was unavailable for 11 (5.9%). Most samples were obtained from primary tumors (*n* = 135, 71.4%), while 48 (25.4%) were from metastatic sites, and 6 (3.2%) lacked specification.

### 3.2. Recurrent Mutations and Copy Number Alterations

**Figure 1:**
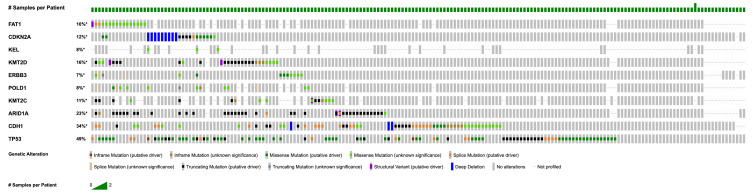
Landscape of genetic alterations across the cohort. Oncoprint chart showing the distribution of the most frequently altered genes. Each column represents a tumor sample, and each row corresponds to a specific gene. Mutation types, including missense, truncating, splice site, and promoter mutations, are color-coded, and amplifications are shown in red, whereas deletions are shown in blue. * Not all samples from the total cohort were profiled.

The ten most frequently mutated genes across the 189 tumor samples are shown in Figure 1. *TP53* was the most commonly altered gene, mutated in 92 samples (48.7%). Other recurrent mutations included *CDH1* (*n* = 59, 31.2%), *ARID1A* (*n* = 40, 21.2%), *KMT2C* (*n* = 16, 8.5%), and *POLD1* (*n* = 14, 7.4%). Additional mutations were observed in *ERBB3*, *KMT2D*, *KEL*, *CDKN2A*, and *FAT1*, each present in approximately 1–7% of samples.

Copy number alterations were profiled in 159 tumors. Frequent amplifications included *CCNE1* (*n* = 13, 8.2%) and *FGFR2* (*n* = 12, 7.6%), with additional gains in *MET* and *MYC* (each *n* = 8, 5.0%) as well as *KRAS* and *ERBB2* (each *n* = 7, 4.4%). Losses were observed in tumor suppressor genes, including *CDKN2A* (*n* = 9, 5.7%), *CDKN2B* (*n* = 8, 5.0%), *CDH1* (*n* = 3, 1.9%), and *PTEN* (*n* = 3, 1.9%). Collectively, these findings highlight the contribution of both recurrent mutations and copy number alterations to the genomic landscape of this cohort.

### 3.3. Analysis by Race and Sex

When stratified by race, multiple genes exhibited mutations exclusively in White patients, while no alterations were observed in Asian, Black, or Other groups (Table 2). For example, *AFDN*, *AFF3*, *BCL9*, *CDX2*, *CLTCL1*, *CREB3L1*, *CREB3L2*, and *DNA2* were each mutated in 2 of 2 White patients tested (100%) and absent in all non-White patients. Similar White-only enrichment was also noted in *KCNJ5*, *LIG1*, *MLF1*, and *ZNF384*. These associations were highly significant (*p* < 1 × 10^−10^, *q* < 1 × 10^−10^). While small sample sizes warrant caution, the consistency of White-only alterations across multiple genes suggests possible race-specific patterns that may inform future studies of disease biology and disparities; however, these are hypothesis-generating only given the 2/2 denominators and cohort imbalance.

When stratified by sex, distinct patterns of genomic alterations were observed. *CCNE1* alterations (amplifications) were identified in 12 of 76 tested females (15.8%) compared with only 1 of 95 tested males (1.0%), representing a significant female enrichment (*p* = 2.83 × 10^−4^) but not after FDR (*q* = 0.205). *ZFHX4* alterations were present in 2 of 6 tested males (33.3%) and absent in females. *AGO2* mutations occurred in 6 of 44 tested females (13.6%) compared with 1 of 58 tested males (1.7%). *ERBB3* mutations were found in 10 of 79 tested females (12.7%) versus 4 of 99 tested males (4.0%). Collectively, these results suggest potential sex-specific mutation patterns, with *CCNE1* showing the strongest female-predominant enrichment.

### 3.4. Pairwise Analysis of Mutations

Pairwise analysis revealed distinct patterns of co-occurrence and mutual exclusivity among frequently altered genes. *POLD1* mutations significantly co-occurred with *KMT2D* (*p* < 0.001, *q* < 0.001) and with *ARID1A* (*p* < 0.001, *q* < 0.001). *ARID1A* and *KMT2D* also showed significant co-occurrence (*p* < 0.001, *q* = 0.007). Additional co-occurrence was observed between *POLD1* and *KEL* (*p* = 0.005) and between *POLD1* and *FAT1* (*p* = 0.008), as well as between *KMT2D* and *FAT1* (*p* = 0.016), *KMT2D* and *KMT2C* (*p* = 0.017), and *ARID1A* and *FAT1* (*p* = 0.024).

Conversely, *TP53* demonstrated mutual exclusivity with *ARID1A* (*p* = 0.029) and *CDH1* (*p* = 0.041). Together, these findings highlight cooperative interactions between *POLD1*, *ARID1A*, and *KMT2D*, while suggesting that *TP53* may drive tumorigenesis through an independent, mutually exclusive pathway.

### 3.5. Primary and Metastatic Tumors

For this analysis, 135 primary and 48 metastatic tumor samples were compared to evaluate shifts in the genomic landscape with disease progression. A large number of alterations were enriched exclusively in primary tumors, including *AFDN*, *AFF3*, *BCL9*, *CDX2*, *CLTCL1*, *CREB3L1*, *CREB3L2*, *DNA2*, *FNBP1*, *KCNJ5*, *LIG1*, *MLF1*, *NDRG1*, *PBX1*, *PCM1*, *PER1*, *STIL*, *THRAP3*, *ZNF384*, and others (all *p* = 8.50 × 10^−4^, *q* = 0.0224). These genes were mutated in 2 of 2 primary samples tested (100%) and were absent in metastatic tumors, suggesting they may represent early events in tumor development. However, these signals likely reflect complete separation from tiny denominators and require replication. In contrast, *CDH1* mutations were more frequent in metastases (23 of 47, 48.9%) compared with primary tumors (39 of 132, 29.6%; *p* = 0.0205). *MLH1* alterations were also enriched in metastases (4 of 47, 8.5%) compared with primaries (2 of 134, 1.5%; *p* = 0.0402). While *ATM* mutations were observed only in primary tumors (12 of 129, 9.3%), this difference was not statistically significant. Together, these results indicate that distinct sets of genes are preferentially altered in primary versus metastatic disease, with a broad array enriched in primary tumors and *CDH1* and *MLH1* emerging as significant metastatic-enriched alterations.

## 4. Discussion

### 4.1. Study Overview and Genomic Landscape

This study utilized the AACR Project GENIE repository to analyze the molecular profile of poorly differentiated carcinoma of the stomach (PGC) and uncover underlying tumorigenic mechanisms to advance knowledge and improve diagnostic strategies. Analysis across varying patient subgroups revealed notable patterns in genetic mutation with regard to PGC tumor progression. Specifically, the gene *TP53* displayed high levels of mutation (92/189 samples), consistent with established data [18,19].

### 4.2. Race- and Sex-Stratified Mutational Patterns

When stratified by race, a subset of genes appeared to be altered exclusively in White patients, while no mutations were detected in Asian, Black, or other racial groups. Specifically, *AFDN*, *AFF3*, *BCL9*, *CDX2*, *CLTCL1*, *CREB3L1*, *CREB3L2*, and *DNA2* each demonstrated mutations in 2 of 2 White patients tested and were absent in all non-White cases. Additional genes, including *KCNJ5*, *LIG1*, *MLF1*, and *ZNF384*, exhibited similarly restricted patterns, with statistically significant enrichment (*p* < 1 × 10^−10^, *q* < 1 × 10^−10^).

Although these findings were highly significant numerically, the extremely small sample sizes limit biological interpretation. It is unlikely that these mutations are truly exclusive to White patients; rather, they are likely to reflect sampling limitations and demographic imbalances within the GENIE dataset, which is known to overrepresent patients of European ancestry [20,21,22]. Taken together, these patterns likely reflect demographic and sampling biases rather than ancestry-linked genomic drivers. Moreover, current literature does not support race-specific enrichment of these genes in gastric cancer [23], further suggesting that these observations may represent statistical artifacts or cohort effects rather than true biological differences. Prior studies have emphasized that disparities in genomic databases can influence apparent race-associated mutation frequencies, often due to uneven enrollment and sequencing coverage rather than ancestry-linked biology.

Nonetheless, the observation that multiple genes displayed consistent alteration patterns within White patients may warrant future exploration in larger, racially diverse gastric cancer cohorts. Investigating whether these genes show reproducible patterns across ancestries could help clarify whether subtle ancestry-linked genomic modifiers contribute to the molecular heterogeneity of poorly differentiated gastric carcinoma.

When examining genomic alterations by biological sex, a genetic discrepancy was evident. Mutations in *CCNE1* occurred primarily in females, with very few in the male sample population. Specifically, *CCNE1* alterations (amplifications) appeared in 12/76 (15.8%) tested female patients compared to 1/95 (1.0%) male patients, demonstrating significance. These findings are supported by current research, as mutations and overexpression of *CCNE1* are typically linked to cancers of the female reproductive system [24,25]. Mutations in *ZFHX4*, *AGO2*, and *ERBB3* were also examined for demographic discrepancies. *ZFHX4* demonstrated a male-skewed pattern (present in 2/6 tested males and absent in females), while *AGO2* and *ERBB3* appeared more common in females; however, none of these differences retained significance after adjustment. Overall, these findings suggest patterns in sex-related mutations, with *CCNE1* showing the strongest female enrichment.

### 4.3. Core Mutational Findings and Pathway Involvement

Corresponding with previous research, our PGC cohort demonstrated substantial genetic heterogeneity with multiple recurrent mutations [26,27]. Among the 189 tumor samples analyzed, five genes exhibited notable mutation frequencies: *TP53* (48.7%), *CDH1* (31.2%), *ARID1A* (21.2%), *KMT2C* (8.5%), and *POLD1* (7.4%). Other mutations were identified in *ERBB3*, *KMT2D*, *KEL*, *CDKN2A*, and *FAT1*, each present in 1–7% of cases.

These findings are consistent with prior large-scale studies of gastric carcinoma. *TP53* is the most frequently mutated gene in gastric cancer, with reported rates ranging from 42% to 63% across major cohorts, including poorly differentiated and signet-ring cell subtypes [28]. *CDH1* mutations are found in approximately 10–32% of diffuse-type and poorly cohesive gastric cancers, where they are linked to loss of cell adhesion and aggressive clinical behavior [29]. *ARID1A* mutations occur in roughly 21–27% of cases and are associated with distinct molecular subtypes and immunogenic features [30]. *KMT2C* alterations are reported in 8–10% of gastric cancers, defining a subset with potential implications for therapeutic response [31]. *POLD1* mutations are less common but have been observed in 7–8% of cases, particularly within hypermutated or microsatellite-stable cohorts [32]. Collectively, these parallels reinforce that our cohort captures the core genomic architecture characteristic of poorly differentiated gastric carcinoma.

Copy number analysis similarly reflected previously published patterns [33,34,35]. Amplifications in *CCNE1* (8.2%) and *FGFR2* (7.6%) occurred at frequencies nearly identical to those reported in prior gastric cancer cohorts [34], highlighting their recurrent involvement in cell-cycle regulation and receptor tyrosine kinase signaling. Gains in *MET*, *MYC*, *KRAS*, and *ERBB2* further support established oncogenic drivers of gastric carcinoma, while deletions in tumor suppressors such as *CDKN2A*, *CDH1*, and *PTEN* align with known mechanisms of unchecked proliferation and invasion.

*TP53* is one of the most commonly mutated genes in cancer, consistent with the central role of the p53 pathway. p53 functions as a transcription factor that prevents the propagation of cells with damaged DNA by activating genes involved in cell cycle arrest and apoptosis, reinforcing its reputation as a major tumor suppressor [36]. *CCNE1* also plays a central role in carcinogenesis through regulation of the G1–S cell cycle transition. Overexpression of *CCNE1* can lead to aggressive tumor growth and genomic instability, making it a target for multiple cancer therapies [37,38]. In PGC, both of these genes are highly significant due to their involvement in cell cycle and tumor regulation. Prior studies have also demonstrated high rates of co-occurrence between *TP53* and *CCNE1* [34]. Another frequently altered pathway in PGC involves *ARID1A*, which functions in DNA repair. Loss of *ARID1A* is frequently observed in gastric tumors and may serve as a target for personalized therapy [39].

### 4.4. Co-Occurrence and Mutual Exclusivity Patterns

Pairwise analysis revealed distinct patterns of co-occurrence and mutual exclusivity. The strongest co-occurring gene pairs were *POLD1*–*KMT2D* (*p* < 0.001, *q* < 0.001), *POLD1*–*ARID1A* (*p* < 0.001, *q* < 0.001), and *ARID1A*–*KMT2D* (*p* < 0.001, *q* = 0.007). Additional co-occurrences included *POLD1*–*KEL* (*p* = 0.005), *POLD1*–*FAT1* (*p* = 0.008), *KMT2D*–*FAT1* (*p* = 0.016), *KMT2D*–*KMT2C* (*p* = 0.017), and *ARID1A*–*FAT1* (*p* = 0.024). These patterns are consistent with findings in other cancers, such as ovarian cancer, where *ARID1A* and *KMT2D* co-occur in approximately 18% of cases [40]. Additional research supports co-occurrence between *POLD1* and *ARID1A* in salivary carcinoma [41]. The co-occurrence of *POLD1* and *KMT2D* is likewise supported by literature showing implications for improved clinical classification and identification of *POLE*-mutated tumors [42]. While these patterns of co-occurrence are well documented in other cancers, they have not been extensively reported for PGC. Our findings suggest that similar molecular relationships—particularly among *POLD1*, *KMT2D*, and *ARID1A*—may also be present in PGC, indicating shared mechanisms of genomic alteration across cancer types.

In contrast, *ARID1A* demonstrated mutual exclusivity with *CDH1* (*p* = 0.029) and *TP53* (*p* = 0.041). This finding is consistent with prior research and may have implications for therapeutic targeting of *ARID1A*-deficient cancer cells [39,43]. These mutually exclusive patterns suggest that *ARID1A* and *CDH1* may form a synthetic lethal pair, potentially informing the development of targeted therapies for gastric carcinoma in the future.

### 4.5. Primary Versus Metastatic Differences and Clinical Correlates

Comparison of primary and metastatic PGC samples revealed that numerous alterations were enriched exclusively in primary tumors (*AFDN*, *AFF3*, *BCL9*, *CDX2*, *CLTCL1*, *CREB3L1*, *CREB3L2*, *DNA2*, *FNBP1*, *KCNJ5*, *LIG1*, *MLF1*, *NDRG1*, *PBX1*, *PCM1*, *PER1*, *STIL*, *THRAP3*, *ZNF384*, and others; all *p* = 8.50 × 10^−4^, *q* = 0.0224). These genes were mutated in 2/2 primary samples tested (100%) and were completely absent in metastatic tumors, suggesting that they may play roles in early tumor development. While broadly consistent with prior reports, the very small sample size makes these findings difficult to validate [44]. Conversely, *CDH1* mutations were more frequent in metastatic tumors (23 of 47, 48.9%) than in primary tumors (39 of 132, 29.6%; *p* = 0.0205). *MLH1* mutations were also more common in metastases (4 of 47, 8.5%) than in primary tumors (2 of 134, 1.5%; *p* = 0.0402). These findings are consistent with reports suggesting that *CDH1* RNA may serve as a biomarker for identifying *CDH1*-related carcinomas [45]. Similarly, *MLH1* alterations may hold therapeutic relevance, as *MLH1* deficiency is associated with chemoresistance in several cancers, including PGC, highlighting its potential as an indicator for alternative treatment approaches [46,47]. *CDX2* expression also warrants attention, as previous studies have shown that *CDX2*-positive tumors exhibit increased sensitivity to 5-FU–based therapy, suggesting its possible use as a biomarker for guiding treatment in “cancer of unknown primary (CUP)” [48]. This therapeutic relevance could extend to PGC, where primary tumors show high rates of *CDX2* alteration.

However, these primary versus metastatic comparisons should not be interpreted as reflecting true evolutionary tumor progression. The AACR GENIE database does not capture site-specific metastatic information (e.g., lymph node, peritoneum, liver), nor does it include paired primary–metastatic samples from the same patient. As a result, differences observed across sample types likely reflect cross-sectional cohort effects rather than longitudinal genomic evolution.

### 4.6. Limitations

This study includes several limitations. Firstly, the AACR Project GENIE database lacks multiple data facets—including transcriptomic, miRNA, and DNA methylation profiles—which restrict biological interpretation. The absence of transcriptomic data precluded correlating mutational status with downstream pathway activation or gene expression changes, which is particularly important in PGC, where high rates of co-occurrence were observed. Similarly, the lack of miRNA and methylation data hindered the evaluation of epigenetic mechanisms and tumor-suppressive regulation in PGC pathogenesis and therapeutic response.

Secondly, GENIE does not provide treatment or outcome information, limiting analyses linking mutational profiles to therapy response, disease progression, or survival endpoints. This also precluded assessment of therapy-induced genomic alterations that may confound observed differences between primary and metastatic tumors.

Thirdly, variability among participating centers and sequencing platforms introduces potential technical inconsistencies and bias in mutation frequency estimates, and the absence of serially collected patient-matched samples limits the ability to distinguish early driver mutations from later-acquired passenger events during tumor evolution. Furthermore, the relatively small cohort size reduced statistical power to establish robust associations between specific genetic alterations and clinical or pathological features; particularly small denominators (e.g., 2/2 cases in some metastatic versus primary and race-stratified comparisons) should therefore be interpreted as hypothesis-generating rather than definitive and may reflect the rarity of specimens or limited participation. Additionally, inclusion of a small subset of related samples (e.g., multiple tumors from the same patient) cannot be fully excluded and could introduce minimal, though likely negligible, bias.

Fourth, GENIE does not uniformly annotate gastric histologic patterns (e.g., tubular, signet-ring, mucinous) or Laurén subtype (intestinal vs diffuse/poorly cohesive), limiting subtype-specific analyses. Likewise, the absence of immunohistochemical data prevented correlation of genetic alterations with protein-level or immune-related expression patterns.

Lastly, the heterogeneity in bioinformatics, data processing, and reporting, and the deficient inclusion of detailed data from the different participants in this database are significant handicaps that may have influenced the results. Unknown details about the participants in particular may skew data if their conditions affect the test results, leading to inaccurate conclusions.

## 5. Conclusions

This study provides valuable insight into the mutational spectrum of PGC, reinforcing the significance of established oncogenic pathways such as *TP53*, *CCNE1*, and *ARID1A*, while revealing potential novel genomic targets that merit further exploration in more comprehensively annotated cohorts. Future investigations should focus on larger, independent cohorts with robust clinical annotation to validate these findings and assess their prognostic and therapeutic implications. Such efforts will facilitate the translation of genomic insights into improved diagnostic, therapeutic, and prognostic strategies for patients with PGC.

## Figures and Tables

**Table 1:** Demographic characteristics of the study cohort. Distribution of sex, age, ethnicity, race, and sample type among patients with PGC (*n* = 188) with reported sample types (*n* = 189). Percentages are calculated based on available data.

Demographics	Category	*n* (%)
Sex	Female	102 (54.26%)
	Male	82 (43.62%)
Age category	Adult	187 (99.47%)
	Pediatric	1 (0.53%)
Ethnicity	Non-Hispanic	136 (72.34%)
	Hispanic	34 (18.08%)
	Unknown/Not Collected	18 (9.57%)
Race	White	115 (61.17%)
	Black	21 (11.17%)
	Asian	19 (10.11%)
	Other	18 (9.57%)
	Unknown	11 (5.85%)
Sample Type ^1^	Primary	135 (71.43%)
	Metastasis	48 (25.40%)
	Not Collected/Unspecified	6 (3.17%)

^1^ Sample type *n* *=* 189.

**Table 2:** Enrichment of specific gene mutations by race and sex. Distribution of gene alterations stratified by race (White vs. non-White) and sex (male vs. female). *p*-values and *q*-values are shown for all comparisons.

Gene (Chi-Squared)	White (*n*, %)	Non-White (*n*, %)	*p*-Value	*q*-Value
*AFDN*	2 (100.0%)	0 (0.00%)	<1 × 10^−10^	<1 × 10^−10^
*AFF3*	2 (100.0%)	0 (0.00%)	<1 × 10^−10^	<1 × 10^−10^
*BCL9*	2 (100.0%)	0 (0.00%)	<1 × 10^−10^	<1 × 10^−10^
*CDX2*	2 (100.0%)	0 (0.00%)	<1 × 10^−10^	<1 × 10^−10^
*CLTCL1*	2 (100.0%)	0 (0.00%)	<1 × 10^−10^	<1 × 10^−10^
*CLYBL*	2 (100.0%)	0 (0.00%)	<1 × 10^−10^	<1 × 10^−10^
*CREB3L1*	2 (100.0%)	0 (0.00%)	<1 × 10^−10^	<1 × 10^−10^
*CREB3L2*	2 (100.0%)	0 (0.00%)	<1 × 10^−10^	<1 × 10^−10^
*CRTC3*	2 (100.0%)	0 (0.00%)	<1 × 10^−10^	<1 × 10^−10^
*DNA2*	2 (100.0%)	0 (0.00%)	<1 × 10^−10^	<1 × 10^−10^
*FNBP1*	2 (100.0%)	0 (0.00%)	<1 × 10^−10^	<1 × 10^−10^
*KCNJ5*	2 (100.0%)	0 (0.00%)	<1 × 10^−10^	<1 × 10^−10^
*KDSR*	2 (100.0%)	0 (0.00%)	<1 × 10^−10^	<1 × 10^−10^
*LIG1*	2 (100.0%)	0 (0.00%)	<1 × 10^−10^	<1 × 10^−10^
*MLF1*	2 (100.0%)	0 (0.00%)	<1 × 10^−10^	<1 × 10^−10^
*NDRG1*	2 (100.0%)	0 (0.00%)	<1 × 10^−10^	<1 × 10^−10^
*NIN*	2 (100.0%)	0 (0.00%)	<1 × 10^−10^	<1 × 10^−10^
*PBX1*	2 (100.0%)	0 (0.00%)	<1 × 10^−10^	<1 × 10^−10^
*PCM1*	2 (100.0%)	0 (0.00%)	<1 × 10^−10^	<1 × 10^−10^
*PDE4DIP*	2 (100.0%)	0 (0.00%)	<1 × 10^−10^	<1 × 10^−10^
*PER1*	2 (100.0%)	0 (0.00%)	<1 × 10^−10^	<1 × 10^−10^
*STIL*	2 (100.0%)	0 (0.00%)	<1 × 10^−10^	<1 × 10^−10^
*THRAP3*	2 (100.0%)	0 (0.00%)	<1 × 10^−10^	<1 × 10^−10^
*ZNF384*	2 (100.0%)	0 (0.00%)	<1 × 10^−10^	<1 × 10^−10^
**Gene (Chi-Squared)**	**Male (** ***n*** **, %)**	**Female (** ***n*** **, %)**	***p*** **-value**	***q*** **-value**
*CCNE1*	1 (1.05%)	12 (15.79%)	2.883 × 10^−4^	0.205
*ZFHX4*	2 (33.33%)	0 (0.00%)	3.918 × 10^−3^	1.00
*AGO2*	1 (1.72%)	6 (13.64%)	0.0405	1.00
*ERBB3*	4 (4.04%)	10 (12.66%)	0.0482	1.00

## Data Availability

The data presented in this study are available from the AACR GENIE Database at https://genie.cbioportal.org/ (accessed on 12 August 2025).

## Abbreviations

The following abbreviations are used in this manuscript:

PGC	poorly differentiated carcinoma of the stomach
AACR	American Association for Cancer Research
miRNA	micro-RNA
5-FU	5-fluorouracil
TP53	tumor protein p53
CDH1	cadherin 1
ARID1A	AT-rich interactive domain 1A
KMT2C	lysine (K)-specific methyltransferase 2C
POLD1	DNA polymerase delta1, catalytic subunit
ERBB3	Erb-b2 receptor tyrosine kinase 3
KMT2D	lysine methyltransferase 2D
KEL	Kell metallo-endopeptidase
CDK2NA	cyclin-dependent kinase inhibitor 2A
FAT1	FAT atypical cadherin 1
CCNE1	cyclin E1
FGFR2	fibroblast growth factor receptor 2
MET	MET proto-oncogene, receptor tyrosine kinase
MYC	MYC proto-oncogene, bHLH transcription factor
KRAS	Kirsten rat sarcoma viral oncogene homolog
ERBB2	Erb-b2 receptor tyrosine kinase 2
CDKN2B	cyclin-dependent kinase inhibitor 2B
PTEN	phosphatase and tensin homolog
MLH1	MutL homolog 1
ATM	ATM serine/threonine kinase
BRCA2	BRCA2, DNA repair-associated
TNM	tumor node metastasis
LOH	loss of heterozygosity
CpG	cytosine, guanine
APC	adenomatous polyposis coli
DCC	deleted in colorectal cancer
GATK	genome analysis toolkit
ANNOVAR	ANNOtate VARiation
PIK3CA	phosphatidylinositol-4,5-biphosphate 3-kinase catalytic subunit alpha
CNAs	copy number alterations
TMB	tumor mutational burden
VAF	variant allele frequency
VUS	variants of uncertain significance
MAF	mutation annotation format
FDR	false discovery rate
ZFHX4	zinc finger homeobox 4
AGO2	argonaute 2, RISC catalytic component
AFDN	afadin, adherens junction formation factor
AFF3	AF4/FMR2 family, member 3
BCL9	B-cell CLL/lymphoma 9
CDX2	caudal type homeobox 2
CLTCL1	clathrin, heavy chain-like 1
CREB3L1	cAMP-responsive element binding protein 3-like 1
CREB3L2	cyclic AMP-responsive element-binding protein 3-like 2
DNA2	DNA replication helicase/nuclease 2
KCNJ5	potassium inwardly rectifying channel subfamily J member 5
LIG1	DNA ligase 1
MLF1	myeloid leukemia factor 1
ZNF384	zinc finger protein 384
NDRG1	N-myc downstream-regulated gene 1
PBX1	pre-B-cell leukemia transcription factor 1
PCM1	pericentriolar material 1
PER1	period circadian regulator 1
PTEN	phosphatase and tensin homolog deleted on chromosome 10
MYC	MYC proto-oncogene, bHLH transcription
ATP	adenosine triphosphate
NDRG1	N-myc downstream regulated 1
STIL	SCL/TAL1 interrupting locus
THRAP3	thyroid hormone receptor-associated protein 3
CUP	cancer of unknown primary
